# Comparison of Postoperative Opioid Consumption and Pain Scores in Primary Versus Repeat Cesarean Delivery in Opioid Naïve Patients

**DOI:** 10.3390/jcm8122221

**Published:** 2019-12-16

**Authors:** Amanda Chao, Ioana Pasca, Matthew Alschuler, Jay Lee, Michelle Woodfin, Justin Pugh, Briahnna Austin, Mark Ringer, Davinder Ramsingh

**Affiliations:** Department of Anesthesiology, Loma Linda University Medical Center, 11234 Anderson Street, MC-2532-D, Loma Linda, CA 92354, USA; ATChao@llu.edu (A.C.); IPasca@llu.edu (I.P.); MAlschuler@llu.edu (M.A.); JAYLee@llu.edu (J.L.); MWoodfin@llu.edu (M.W.); jpugh@llu.edu (J.P.); BRAustin@llu.edu (B.A.); mringer@llu.edu (M.R.)

**Keywords:** cesarean delivery, pain management, primary vs. secondary cesarean delivery, opioids post-cesarean delivery

## Abstract

Background: Cesarean deliveries represent a large percentage of deliveries worldwide. Patients undergoing repeat cesarean deliveries are known to have increased risks for surgical complications. However, little is known regarding potential differences in pain. We sought to compare postoperative opioid consumption and pain scores in opioid naïve patients undergoing primary versus repeat non-emergent cesarean delivery. Methods: This was a retrospective cohort study. Patient inclusion criteria included: having a non-emergent cesarean delivery, receiving a spinal procedure for surgical anesthesia without general anesthesia, and following the same postoperative pain management protocols. Exclusion criteria included: history of opioid tolerance, illicit drug use, or prior, non-obstetric, major abdominal surgery. The primary outcome marker was total morphine equivalents consumed 0–72 h post-procedure compared between the primary versus repeat cesarean delivery groups. Secondary outcome markers were opioid consumption and pain scores in 24-h period increments for the first 72 h postoperatively. Results: 1617 patients were screened. 217 primary and 377 repeat cesarean deliveries met criteria for comparison. Reduced opioid consumption was demonstrated for the total opioid consumption 0–72 h for the repeat cesarean delivery group (median = 35) compared to the primary cesarean delivery group (median = 58), *p* = 0.0005. When divided into 24-h periods, differences were demonstrated for the 24–48 and 48–72 h periods but not the 0–24 h period. Pain scores did not differ statistically. Conclusion: Opioid naïve obstetric patients who undergo non-emergent repeat cesarean delivery demonstrate lower opioid consumption in the postoperative period. Providers should be aware of this potential difference in order to better educate patients and provide adequate pain management. Highlights: The study reviewed differences in opioid consumption between primary and repeat cesarean deliveries. All patients received the same protocol for spinal dosage and pain management. Repeat cesarean deliveries were associated with lower opioid consumption.

## 1. Introduction

There are 1.23 million cesarean deliveries (CD) performed in the United States each year, accounting for 32% of all deliveries [1]. There is a multitude of factors to explain this significant percentage, including change in risk profiles, maternal request, and social and cultural elements [2]. Often after a patient undergoes a primary CD she will receive a repeat CD for future deliveries, with 82% of primary CD patients undergoing a repeat CD [1]. It is known that the operative risk for the repeat CD patient is viewed differently compared to primary CD given the increased risk for scar tissue, bladder/bowel injuries, intraoperative bleeding, and abnormal placentation [2]. However, in terms of pain management, the primary vs repeat CD are often viewed similarly. Pain post CD is an expected event, like all operative procedures, and can impact the recovery of the mother. [3,4,5]. Little is known about the possible differences in postoperative opioid consumption and pain between primary versus repeat CD.

Modern practitioners must be judicious in their opioid administration as one in 300 CD patients become chronic opioid users [6]. A statement from the American College of Obstetricians and Gynecologists (ACOG) advises a stepwise approach to reduce opioid consumption [7]. However, it is still common for anesthesiologists and obstetricians to prescribe opioids postoperatively, as severe pain after CD is a predictor for poor outcomes including chronic pain, post-partum depression, and difficulty with breastfeeding and infant care [3,4,5]. Given this disconnect between recommended guidelines and current clinical practice, identifying factors that may link to higher pain scores and opioid consumption is of particular importance [8,9].

To date, no study has investigated whether there are differences in acute (0–24 h) to subacute (24–72 h) postoperative pain scores and opioid consumption between primary versus repeat CD. One might suspect that repeat CD patients consume more opioids due to scar hyperalgesia, which has been associated with increased pain scores in these patients [10]. Alternatively, one might suspect that repeat CD patients consume fewer opioids, as previous studies have demonstrated repeat CD patients to be at lower risk for chronic pain development than primary CD patients [5]. To gain further insight, we sought to evaluate the postoperative opioid consumption and pain scores in opioid naïve American Society of Anesthesiology Physical Status Classification System type II patients undergoing primary versus repeat, non-emergent CD. The primary outcome marker was total opioid consumption 0–72 h postoperatively. Secondary outcome markers were opioid consumption in 24 h period increments and pain scores in 24 h period increments for the first 72 h postoperatively.

## 2. Materials and Methods

The methods are presented following the STrengthening the Reporting of OBservational studies in Epidemiology (STROBE) checklist methodology [11].

### 2.1. Study Design

This study was approved by the Loma Linda University Medical Center Institutional Review Board (IRB # 5170140) as a retrospective cohort study. The Institutional Review Board waived the requirement for written informed consent given the retrospective chart review study design. 

### 2.2. Participants

The cohort included all obstetric patients who underwent non-emergent CD between 2015 and 2017 at a tertiary academic hospital in the United States and received the same intraoperative anesthesia and postoperative pain management. Intrapartum CD were also included in the study. Postoperatively, all patients during the study period received a protocolized pain management strategy. Within 1 h post-surgery all patients were prescribed acetaminophen 1000 milligrams (mg) intravenously (IV) × 1, Toradol 30 mg IV × 1, fentanyl 25 micrograms mcg IV q5 min as needed, “pro re nata" (PRN), and Dilaudid 0.2 mg IV q15 min PRN. Subsequently patients were prescribed acetaminophen 650 mg per os (PO) every 6 h (q6h) scheduled, Toradol 30 mg IV q6h or ibuprofen 600 mg PO q6h scheduled, and oxycodone immediate release 5 mg PO q6h PRN. Scheduled opioids were provided after patient request and the reported failure of PRN dosage. All patients could refuse scheduled pain medication and PRN medications were provided after patient request. Spinal dosage and postoperative pain management protocols were developed from a multidisciplinary committee approval. Subject inclusion criteria were as follows: (1) 18–60 years of age; (2) (ASA) II; (3) underwent primary or repeat CD at study institution; (4) and received a spinal for surgical anesthesia using the following intrathecal medications: 0.15 mg morphine, 20 mcg fentanyl, and 13.5 +/− 1.5 mg hyperbaric bupivacaine; (5) opioid naïve before the CD. Opioid naïve was defined as recommended by the US Food and Drug Administration (FDA) definition as patients who do not meet the below definition of opioid tolerant, and who have not taken opioid doses at least as much as those listed for opioid tolerance for one week or longer. Exclusion criteria were: (1) ASA >2; (2) opioid allergy; (3) taking any home medication of opioid antagonist or opioid agonist/antagonist within the past two years; (4) history of pain disorder or substance abuse; (5) previous history of major abdominal surgery; (6) emergent procedure; or (7) opioid tolerant. Opioid tolerance was defined as recommended by the US Food and Drug Administration (FDA) definition as patients who are taking, for one week or longer, at least: 60 mg oral morphine/day, 25 µg mcg transdermal fentanyl/hour, 30 mg oral oxycodone/day, 8 mg oral hydromorphone/day, 25 mg oral oxymorphone/day or an equianalgesic dose of any other opioid [12]. Patients were excluded from the study if they underwent both primary and repeat CD in this period.

### 2.3. Variables

All variables were regarded as independent and included morphine equivalents and pain scores. Opioids administered for each patient 0–72 h post-procedure were noted and then converted to morphine equivalence as per a previously validated equivalency calculator [13]. Totals were divided into postoperative periods of: 0–24 h, 24–48 h, and 48–72 h. Pain scores were recorded by the postpartum nurses using a unidimensional instrument with a visual vector scale that allows measurement of perceived pain intensity by numbers to quantify. The scale used has 11 points (0 to 10), with point 0 (zero) representing no pain and point 10 (ten) the worst possible pain. The remaining numbers represent the following intensities of pain (1, 2, 3, and 4 = mild; 5 and 6 = moderate; 7, 8, 9, and 10 = severe). Pain scores were collected and averaged over the same intervals used for opioid comparisons.

### 2.4. Data Sources

All information was collected from the review of the electronic medical record (Epic Systems Corporation, Verona, WI, USA). The entirety of the patient’s health record is stored on this platform at the study institution, including clinical notes, diagnostic studies, vital signs, and all patient assessment notes).

### 2.5. Bias 

All data were reviewed and confirmed by two research personnel at different time points to improve accuracy.

### 2.6. Study Size

Based on our institution’s historical distribution of primary to repeat CD, we determined a ratio of repeat to primary of 1.75. This ratio was applied for our power analysis. A significant difference for our primary outcome marker was set, a priori, at 10 mg of morphine equivalents opioid consumption over 72 h. Support for this threshold was based on previous data demonstrating a statistical difference between comparisons within a range of 7 mg to 16 mg [14]. With these assumptions we determined, using the Mann–Whitney (two independent groups) settings with an alpha = 0.05 (two-tailed test), power = 0.90, N2/N1 ratio = 1.75, effect size = 0.3 (difference in mean taken as 10, with pooled standard deviation of 33), and sample size = 193 and 339 for primary and repeat groups respectively (total = 532 subjects). 

### 2.7. Statistical Methods

The primary outcome marker was a comparison of total morphine equivalents consumed 0–72 h post-procedure between the primary versus repeat CD groups. Secondary outcome markers were opioid consumption in 24-h period increments and pain scores in 24-h period increments for the first 72 h postoperatively between primary versus repeat CD. Continuous variables were summarized using both median and interquartile range. The Mann–Whitney test was used for comparisons between continuous variables. A Chi-Squared test was used to analyze count data. All analyses were performed with R. (version 3.5.1, Department of Statistics of the University of Auckland in Auckland, New Zealand.). A spearman rank correlation was performed to evaluate the association between opioid consumption and pain scores.

## 3. Results

**Figure 1:**
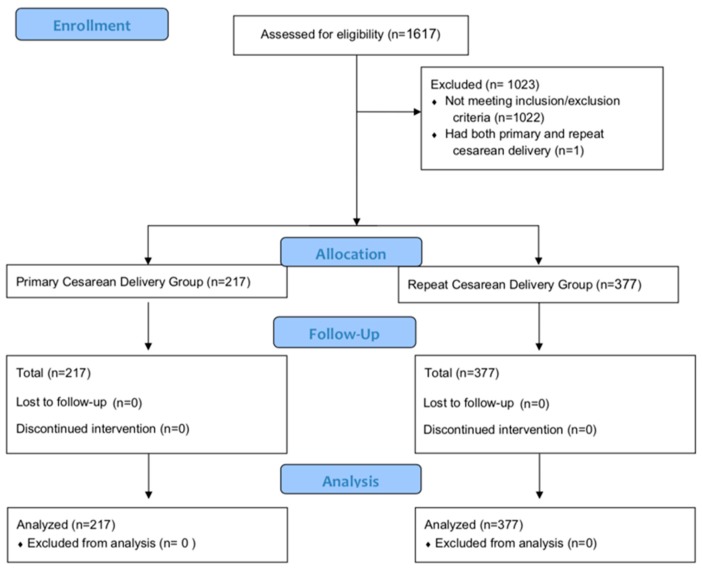
Study CONSORT diagram. (CD = cesarean delivery). After screening 1617 patients via the study inclusion and exclusion criteria, 217 subjects were identified for the primary CD group and 377 for the repeat CD group.

Between the period of August 2015 and May 2017, we screened 1617 patients with 594 patients meeting inclusion/exclusion criteria. Full details regarding patient enrollment are reported in the Consolidated Standards of Reporting Trials diagram (Figure 1). There were 217 subjects in the primary CD group and 377 in the repeat CD group that met all inclusion and exclusion criteria (Figure 1). All patients were admitted for at least 72 h. In addition to the listed exclusion criteria, one patient was excluded for having both her primary and repeat CD in this period (Figure 1). Table 1 reports all patient demographic information. The repeat CD group was found to be older than the primary CD group. For the repeat CD group, 223 (59%) underwent their second CD and 113 (30%) underwent their third CD. Additionally, a large percentage of patients in both groups (Primary CD = 82%, Repeat CD = 76%) were prescribed opioids at discharge (Table 1). 

**Figure 2:**
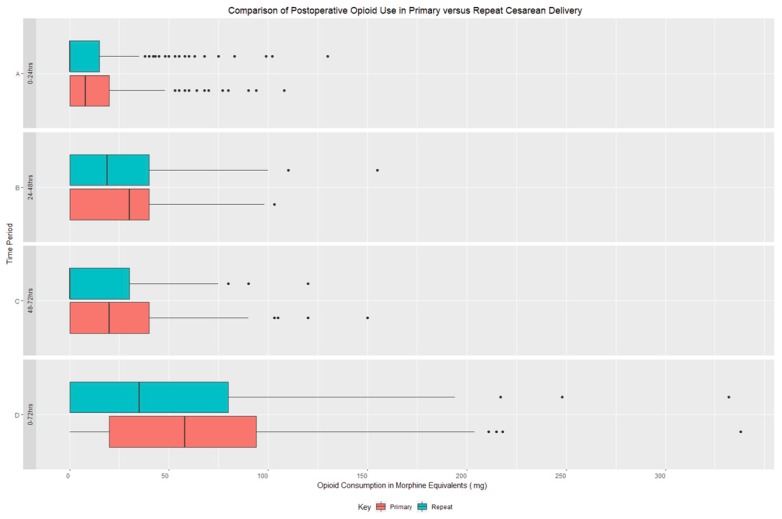
Opioid Administration Post Cesarean (percentage of listed categories of opioid milligram (mg) doses). (CD = cesarean delivery). The repeat CD group had statistically significant reduced total opioid consumption at the 0–72 h, 24–48 h, and 48–72 h post-procedure time intervals (*p* < 0.005).

**Figure 3:**
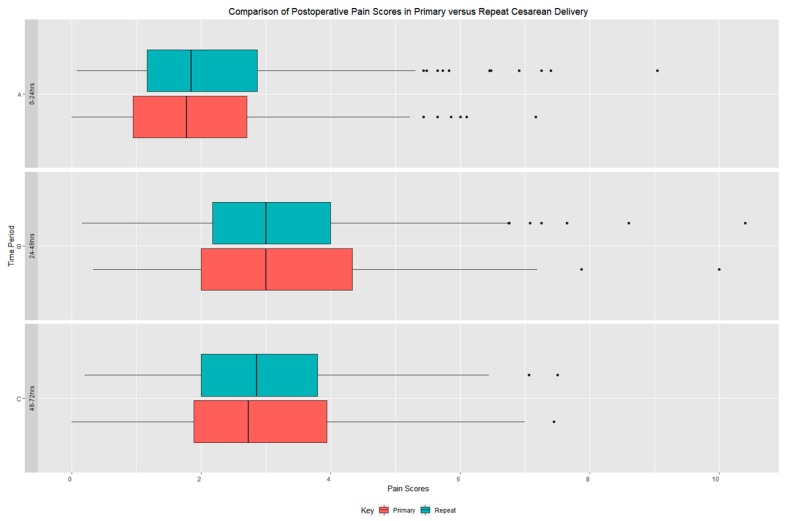
Comparison of pain scores (percentage of pain scores in the identified intervals). (CD = cesarean delivery). No statistically significant differences were observed between the primary and repeat CD groups for all time intervals.

Analyses between the primary CD and repeat CD groups are shown in Table 2. The primary outcome comparison showed statistically significant reduced total opioid consumption 0–72 h post- procedure in patients undergoing repeat CD compared to the primary CD (*p* < 0.005). Additionally, opioid consumption was also significantly reduced at the 24–48 and 48–72 h periods in repeat CD compared to primary CD (*p* < 0.005) (Table 2 and Figure 2). A review of time intervals demonstrated that the 24–48 h period was the period in which opioid consumption was the highest for both groups. A comparison of pain scores aggregated over each time interval did not demonstrate a statistically significant difference (Table 2 and Figure 2). The majority of patients reported pain scores below a scale value of 3/10 for each time interval for both categories with the highest values occurring at the 0–24 h interval (Figure 3). Of note, spearman rank correlation between opioid consumption and pain scores demonstrated R values less 0.43 for all time intervals. Additionally, a large percentage of patients in both groups (Primary CD = 82%, Repeat CD = 76%) were prescribed opioids at discharge (Table 1). Finally, a comparison of non-opioid medication administered showed no difference between the groups.

## 4. Discussion

This study demonstrated a reduction in opioid consumption between patients undergoing repeat versus primary non-emergent CD without showing a difference in postoperative pain scores. These findings are of interest given the high rate of patients undergoing repeat CD procedures. Currently, repeat CD is regarded differently than primary CD in terms of surgical risk but often not regarding postoperative pain management. These findings suggest that postoperative pain management could be regarded differently for patients undergoing repeat CD versus primary CD. 

The development of pain post-CD is widely variable. Multiple factors have been associated with this development including higher body mass index, longer operative time, general anesthesia vs. spinal, age, marital status, hospital stay, and age [15]. This project is the first to suggest a difference in postoperative opioid requirements between patients undergoing primary versus repeat CD. With regard to opioid administration, medical practitioners often prescribe opioids as part of their multimodal analgesic regimen to control postoperative pain after CD [16]. For this study all patients received multi-modal strategies for postoperative pain control, and none received additional regional procedures such as transverse abdominis plane block outside of the spinal anesthetic. 

Indeed, the use of analgesic therapies for the post-CD patient is complex. Additionally, the postpartum period is a known period of anxiety for the mother. Studies have demonstrated anxiety to significantly contribute to increasing pain sensation in women for both non-obstetrical [17,18] and CD [19] postsurgical pain. Koelewijn et al recently performed a prospective cohort study evaluating factors associated with pregnancy-related anxiety. This group demonstrated a lower rate of pregnancy-related anxiety in the multipara vs. nullipara groups [20]. This group also demonstrated a relationship between pregnancy-related anxiety to a higher likelihood to receive pain relief or sedation [20]. These findings support the plausibility that patients undergoing repeat CD may require less postoperative opioids secondary to a reduced state of anxiety associated with the procedure. Additionally, the mother may be worried about these therapies impacting on their capacity to care for or nurse their newborn. It is plausible that the prior experience of patients undergoing a repeat CD may influence their decision to receive opioid therapy secondary to the impact these medications may have on their ability to care for or nurse their newborn. Ultimately this study identifies an association and supports further investigation in the patient management strategies between primary and repeat CD.

This study has several limitations. First, the retrospective study design prevents the standardization of several data points, such as pain score assessment or patient providers. Also, we were not able to differentiate opioids that were ordered per the PRN protocol vs. those provided in a scheduled manner after physician evaluation. Additionally, the comparison groups consisted of different patients and the possibilities of confounders do exist. While this study included only non-emergent, ASA I-II, opioid naïve, CD patients, other confounders are possible. Specifically, the race of the study patients was not captured. For reference, review of our census for labor and delivery admissions for the study time-period demonstrates the following: White Non-Hispanic—46%, Black Non-Hispanic—5.2%, Hispanic—37.5%, Asian and Pacific Is—7%, Other—4.2%. Similarly, there may be a bias in that if a patient experienced significant postoperative pain after their primary CD they may opt out of a repeat CD at the study institution or undergo a trial of labor after CD instead. Importantly, no repeat CD patient was included in the study if they received the primary CD during the study period. Finally, while the study period was targeted such that the spinal dose and postoperative pain orders were standardized, further comparison regarding intraoperative management including providers was not evaluated. It is the authors’ hope that our findings support future prospective studies that account for these confounding variables. 

## 5. Conclusions

This retrospective study demonstrated an association with opioid naïve obstetric patients undergoing non-emergent repeat CD to have lower opioid consumption in comparison to similar patients undergoing primary CD. This information could help providers better educate patients and manage postoperative pain optimally without overprescribing opioids.

## Figures and Tables

**Table 1:** Demographic Data (CD = cesarean delivery, ASA = American Society of Anesthesiologist Classification).

Characteristics Baseline	Measurements	Primary	Repeat	95% CI Estimate (Upper, Lower)	*p* Value
N	Count (Percentage)	217 (36.5%)	377 (63.5%)		
Age (years)	Median (IQR)	30 (7)	31 (7)	−1.99 (−3.00, −2.00)	0.00432
ASA	Median (IQR)	2 (1)	2 (1)	0 (0, 0)	0.7283
CD (#)	Median (IQR)	1 (0)	2 (1)		
CD (#)					
1 2 3 4 5 8	Count (Percentage)	217 (100%) 0 (0%) 0 (0%) 0 (0%) 0 (0%) 0 (0%)	0 (0%) 223 (59%) 113 (30%) 25 (6.7%) 15 (4%) 1 (0.3%)		
Received Prescription for Opioids at Discharge	Count (Percentage)	178 (82%)	286 (76%)		

Demographic details of the patients in the both the primary and repeat CD groups are listed. The frequency of CD procedures and rate in which prescription opioids where provided at discharge are also listed. (IQR = interquartile range, # = number).

**Table 2:** Comparisons of analgesic administration and pain scores.

Characteristics Baseline	Measurments	Primary	Repeat	95% CI Estimate (Upper, Lower)	*p* Value
**Opioids in Morphine Equivalents(mg)**					
0–24 h 24–48 h 48–72 h Total Equivalency(mg)	Median (IQR)	8 (20) 30 (40) 20 (40) 58 (74)	0 (15) 19 (40) 0 (30) 35 (80)	2× 10^−5^ (−3× 10^−5^, 3× 10^−5^) 4 (8× 10^-6^, 10) 4 (4× 10^−5^, 10) 15 (6× 10^−5^, 30)	0.0704 0.0005 0.0005 0.0005
**Non-Opioids-PO Acetaminophen(mg)**					
0–24 h 24–48 h 48–72 h	Median (IQR)	1300 (650) 650 (650) 650 (975)	1300 (650) 650 (650) 1300 (1300)	−5× 10^−5^ (−3× 10^−5^, 1× 10^−5^) −5× 10^-7^ (−4× 10^-6^, 6× 10^−5^) --	0.9778 0.7659 0.8625
**Non-Opioids-IV Acetaminophen (mg)**					
0–24 h 24–48 h 48–72 h	Median (IQR)	1000 (0) -- --	1000 (0) -- --	−8× 10^−5^ (−8× 10^-6^, 5× 10^−5^) -- --	0.9778 -- --
**Non-Opioids-PO Ibuprofen (mg)**					
0–24 h 24–48 h 48–72 h	Median (IQR)	600 (0) 1800 (600) 1800 (600)	600 (600) 1800 (600) 1800 (1200)	−6× 10^-6^ (−3× 10^−5^, 6× 10^−5^) −5× 10^−5^ (−3× 10^-6^, 2× 10^−5^) 3e× 10^−5^ (−2× 10^−5^, 3× 10^-6^)	0.9778 0.9928 0.2682
**Non-Opioids-IV Toradol (mg)**					
0–24 h 24–48 h 48–72 h	Median (IQR)	90 (30) 30 (45) --	90 (60) 30 (7.5) --	5× 10^−5^ (−6× 10^-6^, 3× 10^−5^) 3× 10^−5^ (−7× 10^−5^, 2× 10^−5^) --	0.3748 0.3748 --
**Pain Score**					
0–24 h 24–48 h 48–72 h	Median (IQR)	1.8 (1.7) 3.0 (2.3) 2.7 (2.0)	1.8 (1.7) 3.0 (1.8) 2.9 (1.8)	−0.165 (−0.469, 0.154) 0.0226 (−0.467, 0.529) −8e^−5 (−0.419, 0.403)	0.9778 0.7288 0.7659

Comparisons demonstrated that the repeat CD group had statistically significant reduced total opioid consumption at the 0–72 h, 24–48 h, and 48–72 h post-procedure time intervals (*p* < 0.005). The 24–48 h time interval was the highest period for opioid consumption. Comparison of non-opioid medication administered showed no difference between the groups. Finally, a comparison of pain scores demonstrated no significant difference between groups. (h = hour, mg = milligram, CD = cesarean delivery).
